# StimBlue^+^− mediated modulation of growth and phytochemical traits in *Dasispermum suffruticosum* for sustainable nutrition and health

**DOI:** 10.3389/fnut.2026.1885424

**Published:** 2026-09-07

**Authors:** Zimbini Soganga, Hildegard Witbooi

**Affiliations:** Department of Agronomy, University of Fort Hare, Alice, South Africa

**Keywords:** antioxidant capacity, *Dasispermum suffruticosum*, functional foods, seaweed-derived biostimulants, secondary metabolites, underutilised plants

## Abstract

*Dasispermum suffruticosum* (P. J. Bergius) B. L. Burtt is an aromatic halophyte recognized for its diverse phytochemistry and medicinal plant potential. Despite its potential value, limited scientific information is available regarding its agronomic performance and phytochemical responses. This study investigated the morphological, mineral, and phytochemical responses of *D. suffruticosum* to gradient dosages of StimBlue^+^, a seaweed-derived biostimulant, applied via foliar spray (2.5, 5.0, and 7.5 mL/L) and growing-medium drenching (5.0, 7.0, and 9.0 mL/L), alongside an NPK fertiliser control. Two sequential trials were conducted under semi-controlled glasshouse conditions using a Randomised Complete Block Design (RCBD). Morphological parameters and mineral uptake were assessed over 12 weeks, while phytochemical analyses, including (TPC), and antioxidant capacity (ABTS, FRAP, and DPPH) were conducted for 10 weeks on the three best-performing treatments following the first trial. Growing medium drench at 7.0 mL/L (T5) produced significantly higher stem diameter (1.24 mm), plant height (7.29 cm), number of leaves (8.87), and chlorophyll content (1.30 mg/ m^2^) than the control (*p* ≤ 0.05), while foliar spray at 2.5 mL/L (T1) recorded the highest root development. StimBlue^+^ treatments showed descriptively higher concentrations of several minerals including leaf K, Mg, Ca, Na, Fe and Zn compared with the control. T1 also recorded the highest TPC (20.25 and 8.71 mg GAE/g DW) and ABTS (171.32 and 122.90 μmol TE/g DW), FRAP (110.86 and 63.62 μmol AAE/g DW), DPPH (68.14 and 43.77 μmol TE/g DW), in leaves and roots, respectively. These findings provide preliminary baseline phytochemical and mineral data for *D. suffruticosum* and indicate its potential as a candidate functional food, with low-dose foliar StimBlue^+^ application emerging as a promising treatment warranting further investigation, including replicated trials and targeted bioactive compound characterisation, before nutraceutical application can be confirmed.

## 1 Introduction

Malnutrition remains a major global health burden, with over 820 million people facing food insecurity and malnourishment worldwide (1). The majority of Sub-Saharan African (SSA) regions hunger and low-quality diets contributes to the 11 million annual death cases linked to FI and of these approximately 2 million cases are directly linked to inadequate consumption of fruit and vegetable and South Africa accounts for 25% of the cases. Agricultural intensification aimed at maximising yield has, paradoxically, contributed to declining nutrient density in food crops (2, 3), prompting renewed interest in indigenous and underutilised plant species as sources of functional foods (28).

One such species is *Dasispermum suffruticosum* (P. J. Bergius) B. L. Burtt, an aromatic halophyte endemic to South Africa commonly known as sea parsley, which remains largely absent from scientific literature and commercial exploitation (4, 5). *D. suffruticosum* is one of seven species within the genus *Dasispermum*, belonging to the Apiaceae () family or better known as the parsley/carrot family. Members of this family are widely recognised as rich sources of dietary fibre, mineral elements, and vitamins (A, C, and K) (6), and exhibit extensive phytochemical diversity encompassing alkaloids, polyacetylenes, terpenoids, and phenylpropanoids (29, 30). Despite this potential value, limited scientific information is available regarding its agronomic performance, mineral composition, phytochemical profile in response to any cultivation method. To the best of our knowledge, no peer-reviewed study has investigated these characteristics or evaluated the species’ response to biostimulant application. A systematic search of Scopus, Web of Science and Google Scholar (March 2024–April 2026) using the terms “*Dasispermum suffruticosum*” combined with “mineral,” “phytochemical,” “antioxidant,” “nutraceutical” and “biostimulant” returned no relevant peer-reviewed publications.

Seaweed extract (SWE) biostimulants have been widely reported to improve plant growth, nutrient acquisition and the accumulation of bioactive compounds in numerous cultivated crop species; however, their effects on halophytic, underutilised indigenous species such as *D. suffruticosum* remain largely unexplored. Consequently, StimBlue^+^, a commercial biostimulant derived from the brown macroalga *Macrocystis pyrifera* (31, 53), was selected for evaluation in the present study. StimBlue^+^ contains a diverse range of bioactive constituents, including polysaccharides, polyphenols, pigments, phytohormones, nutrients and proteins, which have been shown to regulate multiple physiological processes associated with plant growth, development, nutrient utilisation, abiotic stress tolerance and crop quality (31, 32).

Moreover, while seaweed-extracted (SWE) biostimulants are well documented to enhance growth and bioactive compound accumulation in numerous conventional crop species, their effects on halophytic, unmanaged indigenous species such as *D. suffruticosum* have not been investigated entirely. Specifically StimBlue^+^, a seaweed extract derived from the brown macroalga *(Macrocystis pyrifera)* (Camus et al., 2018; 31) *was selected as a biostimulant of interest in this study.* These substances comprise diverse components including polysaccharides, polyphenols, pigments, phytohormones, nutrients, and proteins, *which are reported to target* var*ious plant mechanisms to* enhance growth, development, abiotic stress resistance, reproduction, and yield quality (31, 32).

Previous studies have demonstrated the phytostimulatory properties of seaweed extracts, resulting in improved growth and yield across a range of agriculturally important plant species Ali et al. (7). For instance, TAM®, a commercial SWE enhanced the growth, yield, and bioactive chemical contents of *Cucumis sativus, Eruca sativa, Capsicum annuum*, (cucumber, rocket, and hot pepper) (8–10). Comparable physiological improvements have also been reported in *Capsicum annuum*, where biostimulant-treated plants exhibited greater biomass, transpiration and fruit production than untreated plants (33), while Cristiano et al. (34) observed increases of 52% in net photosynthesis, 55% in transpiration, 9% in intracellular CO₂ concentration and 0.8% in stomatal conductance following biostimulant application. In a study carried out by Mannino et al. (10) application of SWE enhanced antioxidant activity in *Solanum lycopersicum* through increased ABTS and DPPH activity. Similarly, the same was later observed by Ashour et al. (8) whereby the antioxidant in tomato fruits increased by 11% (DPPH) and 38% (ABTS) following biostimulant treatment. These responses could be ascribed to the antioxidant compounds and elicitor molecules present in SWE biostimulants that stimulate secondary metabolite biosynthesis and reactive oxygen species scavenging.

Therefore, the present study aimed to characterise the mineral composition, phytochemical profile, and antioxidant capacity of *D. suffruticosum* in response to gradient dosages and application techniques of brown seaweed-extracted (SWE) StimBlue^+^ biostimulant. With the objectives of: (i) determining the effect of its varying application methods and dosages on the mineral uptake, phytochemical accumulation, and antioxidant capacity on *D. suffruticosum* leaf and root tissues, (ii) thus evaluating its potential on *D. suffruticosum* as a candidate crop for functional food and nutraceutical development, based on its bioactive compound profile; and (iii) contributing to food security and dietary diversification for lower socio-economic coastal communities across South Africa.

## 2 Materials and methods

### 2.1 Study site

The present study was conducted under semi-controlled glasshouse conditions at the research facility of the University of Fort Hare, Alice Campus, situated at coordinates 32.78°S, 26.85°E at an altitude of 524 m above sea level. The experimental site falls within the Raymond Mhlaba Local Municipality of the Amathole District Municipality, Eastern Cape Province, South Africa.

### 2.2 Plant collection and preparation

Wild *Dasispermum suffruticosum* plant material was collected on 12 September 2024 from the Walker Bay coastal area in the Western Cape, South Africa (Ethical Clearance: WIT031SSOG01; collection permit No: CN35-28-30733) and transported to the University of Fort Hare semi-controlled glasshouse. On the same day, a preliminary stem-cutting propagation trial was initiated. Following the rooting period, 84 uniformly sized and well-rooted individuals were selected on 2nd June 2025. The plants were transplanted into 4.5 L black polyethylene growing bags filled to 90% capacity with a 1:1 (v/v) coco peat and river sand growing medium and arranged at 1 m and 0.8 m inter and intra-row spacing, respectively. The plants were manually irrigated three times daily using a 2 L watering can, with excess water draining freely through holes at the base of the growing bags, and were allowed a 14-day acclimatisation period prior to treatment application.

### 2.3 Research experimental design and treatment structure

The experiment was arranged in a Randomised Complete Block Design (RCBD) comprising 12 blocks and seven treatments, consisting of six StimBlue^+^ dosage-by-method combinations and one NPK synthetic fertiliser control. Each block contained one experimental unit (a single plant in an individual growing bag) per treatment, such that every treatment was represented once within each block. Replication in the RCBD was achieved through the blocks themselves; accordingly, the design comprised 12 replicates per treatment and 84 experimental units in total (12 blocks × 7 treatments = 84). Treatments comprised six concentrations of liquid StimBlue^+^ biostimulant (manufactured by Kelp Blue Namibia (Pty) Ltd.; constituents presented in Table 1), applied using two delivery methods, together with a powdered NPK synthetic fertiliser (manufactured by InteliGro Crop Solutions (Pty) Ltd) as the conventional control. The delivery methods consisted of foliar spray applications at 2.5, 5.0 and 7.5 mL/L, and growing medium drench applications at 5.0, 7.0 and 9.0 mL/L, with each treatment solution prepared in 1 L of deionised water.

**Table 1:** Two-way ANOVA showing F-ratios from REML mixed model analysis for the effect of treatment, time, and their interaction on vegetative growth parameters of *D. suffruticosum.*

Source of variation	df	Stem diameter (mm)	Leaf height (cm)	No. of leaves	Chlorophyll content(mg/m^2^)
Treatment	6	5.15***	33.49***	58.50***	21.84***
Block	11	2.03*	1.04 ns	1.57 ns	1.70 ns
Weeks	6	69.5***	1529.18***	4162.14***	2409.47***
Treatments* weeks	36	1.36 ns	6.18***	14.94***	4.85***
Residual	462	-	-	-	-
Total	521				

*Re*sidual df = DFDEN and df = degree of freedom.

*p ≤ 0.05, **p ≤ 0.01, ***p ≤ 0.001, n.s. = not significant.

The NPK fertiliser control was prepared in the same manner at a concentration of 2.5 g/L in 1 L of deionised water and applied as a growing medium drench. All treatment solutions were applied bi-weekly from day 15 post-acclimatisation until harvest on 2 September 2025. Foliar solutions were applied uniformly to both the adaxial and abaxial leaf surfaces using a 1 L spray bottle held approximately 2 cm above the plant canopy until the foliage was thoroughly wetted. Drench solutions were applied uniformly to the growing substrate at the base of each plant using a 50 mL beaker. Fresh treatment solutions were prepared immediately before each application. Trial 1 was conducted over 12 weeks to evaluate vegetative growth and mineral nutrient uptake. Based on the morphological, physiological and mineral uptake responses recorded during Trial 1, the three best-performing StimBlue^+^ treatments were selected, together with the NPK fertiliser control, for a second trial conducted from 8 December 2025 to 27 February 2026 to investigate phytochemical compound accumulation. Treatment selection was based on consistent assignment to Tukey–Kramer HSD group “a” across growth parameters, together with superior mineral nutrient uptake. Based on these criteria, foliar spray applications at 2.5 mL/L (T1) and 7.5 mL/L (T3), and the growing medium drench application at 7.0 mL/L (T5), were identified as the best-performing treatments. Trial 2 was also arranged in a Randomised Complete Block Design comprising four blocks and four treatments, with five plants per treatment within each block (4 × 4 × 5, *N* = 80). The treatments were renamed as T1 (foliar spray, 2.5 mL/L), T2 (foliar spray, 7.5 mL/L), T3 (growing medium drench, 7.0 mL/L) and T4 (NPK fertiliser control, 2.5 g/L), with each treatment solution prepared in 1 L of deionised water Table 2.

**Table 2:** F-ratios from REML mixed model analysis for the effect of treatment, time, and their interaction on root length and number of roots of *D. suffruticosum at* initial transplanting (Week 1) and final harvest (Week 12).

Source of variation	df	Root length	No. of roots
Treatment	6	2.4200*	2.2264 ns
Block	11	1.2737 ns	1.0023 ns
Weeks	1	1146.018***	370.1392***
Treatments* weeks	6	2.2850*	1.2690 ns
Error (residual)	77	—	—
Total	101		

*p ≤ 0.05, **p ≤ 0.01, ***p ≤ 0.001, n.s. = not significant.

### 2.4 Growth parameters and nutrient uptake

Growth parameters including, plant height (cm), leaf number, stem diameter (mm), root number and length (cm) were assessed at transplanting and bi-weekly thereafter. Root number and root lengths were measured twice at transplanting and harvest. Chlorophyll content was measured non-destructively using chlorophyll content meter (CCM-300) (Opti-Sciences, United States) and expressed as chlorophyll concentration (mg/m^2^). Readings were taken bi-weekly from the most recently fully expanded leaves from week one to twelve. For mineral uptake analysis, at harvest, plant material was separated into leaf and root fractions, thoroughly rinsed under running tap water to remove surface debris and residual biostimulant, and air-dried at room temperature. Due to the 100 mg fresh weight requirement of the analytical protocol, biological replicates within each treatment were pooled into a single composite sample per tissue fraction, yielding 14 composite samples (seven leaf and seven root) across all seven treatment groups. Composite samples were sealed in polyethylene zip-lock bags, packed under dry ice in an insulated cooler, and transported to BemLab (Pty) Ltd. (Somerset West, Western Cape, South Africa) for elemental analysis. Given the composite sampling approach, mineral data are reported descriptively without inferential statistical analysis.

Total nitrogen (%N) was determined by Dumas dry combustion using a LECO CNS 2000 analyser (LECO Corporation, MI, United States), with a 0.3 g subsample combusted at 1300 °C in a pure oxygen atmosphere and N₂ quantified via thermal conductivity detection (35). Concentrations of P, K, Ca, Mg, Na, Mn, Fe, Cu, Zn, and B were determined by dry ashing at 500 °C, followed by acid dissolution in 20% HCl and quantification by ICP-OES (36, as cited in 37). Mo and S were determined via closed-vessel microwave-assisted nitric acid digestion followed by ICP-OES, owing to the known volatilisation risk of both elements at muffle furnace temperatures (38). All elemental concentrations are expressed as percentage of dry weight (%DW) for macronutrients and mg kg/ DW for micronutrients.

### 2.5 Sample preparation for the determination of total polyphenols and antioxidant activity

At week ten, plants from the four selected treatments were harvested, separated into leaf and root fractions, and thoroughly washed under running tap water. Following air-drying at room temperature, plant material was transferred to a well-ventilated glasshouse and dried for 7 days until fully desiccated. Dried material was subsequently crushed to a fine powder using 1 Peak laboratory blender. They were then sealed in polyethylene zip-lock bags, and submitted to the Oxidative Stress Research Laboratory at the Cape Peninsula University of Technology (CPUT), Bellville, South Africa, for analyses. All the analyses were performed in triplicate to ensure analytical reproducibility.

### 2.6 Determination of total polyphenolic content and antioxidant capacity (FRAP, DPPH, ABTS)

Total polyphenol content was determined using the Folin–Ciocalteu colorimetric method as described by Singleton and Rossi (39), with modifications adapted from Hillis and Swain (40) for microplate analysis, and results were expressed as mg GAE/g dry weight (DW). Ferric reducing antioxidant power (FRAP), based on electron transfer, was measured according to Benzie and Strain (41), and expressed as μmol Trolox equivalents per gram dry weight (μmol TE/g DW). The ABTS radical scavenging activity was determined following Re et al. (42), while DPPH radical scavenging activity was assessed using the method of Brand-Williams et al. (43). Both ABTS and DPPH results were expressed as μmol Trolox equivalents per gram dry weight (μmol TE/g DW).

### 2.7 Statistical analysis

Statistical analyses were conducted using JMP statistical software (version 18, SAS Institute Inc., Cary, NC, United States). Growth parameters measured during the experimental period were analysed using a Restricted Maximum Likelihood (REML) linear mixed-effects model fitted under the Standard Least Squares personality. Treatment, week and the treatment × week interaction were specified as fixed effects, while block and individual plants (Plant_ID) were included as random effects to account for the randomised complete block design and repeated measurements obtained from the same experimental units. Model parameters were estimated using the REML method, with random effects modelled using a variance-components covariance structure. Where significant treatment effects were detected (*p* ≤ 0.05), least-squares means were separated using the Tukey–Kramer Honest Significant Difference (HSD) test. Leaf phytochemical variables, including total phenolic content (TPC), ABTS, FRAP and DPPH, were analysed using one-way analysis of variance (ANOVA), followed by the Tukey–Kramer Honest Significant Difference (HSD) test at a significance level of *α* = 0.05. Mineral composition of leaf and root tissues and the phytochemical composition and antioxidant activity of root tissues were determined from composite samples without biological replication. Consequently, these datasets were analysed descriptively and are presented without inferential statistical testing.

## 3 Results

### 3.1 The effect of different application methods and dosage levels of StimBlue^+^ biostimulant on growth parameters of *D. Suffruticosum*

The REML mixed-model analysis revealed significant treatment effects for most growth parameters evaluated throughout the 12-week experimental period. Week significantly influenced all measured variables (*p* < 0.0001), indicating continuous vegetative development over time. Significant Treatment × Week interactions were observed for plant height, leaf number, chlorophyll content and root length, whereas stem diameter and root number showed no significant interaction with time (Tables 1, 3).

**Table 3:** Effect of StimBlue⁺ application methods and doses on vegetative growth parameters of *D. suffruticosum*.

Treatment	Stem diameter (mm)	Leaf height (cm)	No. of leaves	Chlorophyll content (mg/ m^2^)	No. of roots	Root length (cm)
Control	1.16 ± 0.05abc	6.20 ± 0.08b	7.83 ± 0.10b	1.10 ± 0.01b	4.62 ± 0.21b	5.89 ± 0.19b
T1	1.12 ± 0.05abc	6.98 ± 0.08a	8.87 ± 0.10a	1.25 ± 0.01a	5.58 ± 0.21a	6.73 ± 0.19a
T2	0.96 ± 0.05c	6.22 ± 0.08b	7.08 ± 0.10c	1.13 ± 0.01b	4.83 ± 0.21ab	6.16 ± 0.19ab
T3	1.21 ± 0.05ab	7.03 ± 0.08a	8.73 ± 0.10a	1.27 ± 0.01a	5.00 ± 0.21ab	6.47 ± 0.19ab
T4	1.00 ± 0.05bc	6.28 ± 0.08b	7.17 ± 0.10c	1.18 ± 0.01b	4.88 ± 0.21ab	6.03 ± 0.19ab
T5	1.24 ± 0.05a	7.29 ± 0.08a	8.87 ± 0.10a	1.30 ± 0.01a	5.29 ± 0.21ab	6.43 ± 0.19ab
T6	0.99 ± 0.05c	6.32 ± 0.08b	7.21 ± 0.10c	1.20 ± 0.01b	5.04 ± 0.21ab	6.10 ± 0.19ab
p(*p* ≤ 0.05)	<0.0002	<0.0001	<0.0001	<0.0001	0.0512	0.031

Values represent least squares means ± SE (*n* = 12). Different letters within a column indicate significant differences (Tukey–Kramer HSD, *p* ≤ 0.05).

Different Tukey letters represent significance (*p* ≤ 0.05); same letters represent non-significance (*p* ≤ 0.05).

Overall, StimBlue^+^ promoted vegetative growth compared with the fertiliser control, although the magnitude of the response varied according to application method and dosage. Among the treatments, the 7.0 mL/L growing medium drench treatment (T5) produced the strongest above-ground growth response and recorded the greatest stem diameter, plant height, leaf number and chlorophyll content (Table 3). By contrast, the 5.0 mL/L foliar treatment (T2) and the highest growing medium drench treatment (9.0 mL/L; T6) produced the weakest growth responses among the StimBlue^+^ treatments Table 4

**Table 4:** Comparison of StimBlue application methods on the number of leaves, height and chlorophyll content of *D. suffruticosum*.

Application method	Dose (mL/L or g/L)	No. of leaves (*F* = 58.50***)	Leaf height (cm) (*F* = 33.49***)	Chlorophyll (mg/m^2^) (*F* = 21.84***)
Control	2.5 g/L (T7)	7.83 ± 0.10b	6.34 ± 0.08b	1.18 ± 0.01b
Foliar spray	2.5 mL/L (T1)	8.87 ± 0.10a	6.98 ± 0.08a	1.25 ± 0.01a
	5.0 mL/L (T2)	7.08 ± 0.10c	6.22 ± 0.08b	1.13 ± 0.01b
	7.5 mL/L (T3)	8.73 ± 10a	7.03 ± 0.08a	1.27 ± 0.01a
Drenching	5.0 mL/L (T4)	7.17 ± 0.10c	6.28 ± 0.08b	1.18 ± 0.01b
	7.0 mL/L (T5)	8.87 ± 0.10a	7.29 ± 0.08a	1.30 ± 0.01a
	9.0 mL/L (T6)	7.21 ± 010c	6.32 ± 0.08b	1.20 ± 0.01b
*p*-value <0.0001		<0.0001	<0.0001	<0.0001

Parameters selected based on the highest treatment F-ratios (p ≤ 0.001), values represent least square means ± SE and different letters indicate significant differences (*p* ≤ 0.05).

Different Tukey letters represent significance (*p* ≤ 0.05); same letters represent non-significance (*p* ≤ 0.05).

The 2.5 mL/L foliar treatment (T1) recorded the highest root length and root number; whereas the fertiliser control recorded the lowest values for root growth and chlorophyll content (Table 3.1 and 3). Although treatment effects for root number were not statistically significant for the overall treatment effect, Tukey–Kramer HSD comparisons identified significant differences between T1 and the fertiliser control. Comparison of the two application methods further highlighted differences in growth responses (Table 5). The 7.0 mL/L growing medium drench treatment (T5) recorded the greatest plant height and chlorophyll content, whereas both T5 and the 2.5 mL/L foliar treatment (T1) recorded the highest leaf number.

**Table 5:** Effect of StimBlue⁺ biostimulant on macro-and micro nutrient composition of *D. suffruticosum lea*f and root tissues, expressed as percentage (%) of dry weight (DW).

Plant part	Treatment	N (%)	P (%)	K (%)	Mg (%)	Ca (%)	S (%)	Na (mg/kg)	Fe (mg/kg)	Cu (mg/kg)	Zn (mg/kg)	B (mg/kg)	Mo (mg/kg)	Mn (mg/kg)
Leaves	Control	4.50	0.31	6.37	0.26	2.83	0.59	8,080	198	<1.3	100	26.9	849	266
	T1	3.11	0.20	6.43	0.29	4.33	0.31	6,880	189	2.1	70.2	27.6	4,030	221
	T2	3.10	0.20	6.30	0.28	4.44	***	6,820	225	1.6	57.1	28.9	***	240
	T3	3.69	0.26	6.56	0.27	3.50	0.43	7,610	174	1.7	64.0	23.4	1800	230
	T4	3.09	0.17	5.80	0.28	4.84	0.32	7,600	244	3.5	75.6	26.1	5,210	255
	T5	3.07	0.16	6.90	0.31	4.04	0.31	7,460	236	2.0	57.1	20.1	2,230	247
	T6	3.64	0.16	5.96	0.28	4.52	0.34	8,100	235	2.2	85.5	25.1	2,530	188
Roots	Control	2.83	0.26	3.77	0.73	1.15	***	5,190	784	<1.3	46.6	28.8	***	146
	T1	1.80	0.16	3.05	1.24	1.62	***	7,530	1,410	<1.3	25.5	24.0	***	246
	T2	1.56	0.18	3.38	1.36	2.22	***	8,590	1760	<1.3	<1.3	9.1	***	314
	T3	1.92	0.18	3.92	0.97	1.08	***	7,000	702	<1.3	12.4	17.0	***	180
	T4	1.18	0.17	3.21	1.73	2.48	***	9,190	2,310	<1.3	85.2	24.5	***	292
	T5	2.07	0.18	3.46	1.46	1.78	***	7,960	1800	1.3	26.6	27.3	***	400
	T6	2.41	0.16	3.01	1.27	1.66	***	7,340	1,360	<1.3	28.6	21.8	***	221

*** = not detected (N. D); values reported as <1.3 were below the detection limit, DW, dry weight.

### 3.2 Effect of StimBlue^+^ biostimulant on macro-and micro nutrient composition of *D. Suffruticosum*

The macro and micronutrient concentrations of *D. suffruticosum* leaf and root tissues are presented in. As mineral analysis was conducted on composite samples for each treatment, the results are presented descriptively in Table 6. Overall, the fertiliser control recorded the highest N concentrations in both leaves (4.50%) and roots (2.83%). In contrast, StimBlue^+^ treatments generally enhanced the accumulation of Ca, K, and Mg, with T4 recording the highest leaf calcium concentration (4.84%) and T5 the highest leaf potassium concentration (6.90%). Among the micronutrients, root Fe accumulation was generally greater under StimBlue^+^ treatments than in the control, reaching 2,310 mg k/g in T5 compared with 784 mg k/g in the control. Leaf Mo concentrations were also higher in T1 and T4 StimBlue^+^ treatments than in the control, although molybdenum was not detected in root tissues or in the leaves of T2 (Table 5).

**Table 6:** Total polyphenol content of *D. suffruticosum* root tissue in response to StimBlue⁺ application methods and doses.

Treatments	Treatment description	TPC (mg GAE/g DW)
T1	Foliar spray 2.5 mL/L	10.95 ± 0.74
T2	Foliar spray 7.5 mL/L	9.85 ± 0.15
T3	Soil drench 7.0 mL/L	10.41 ± 0.25
T4	Fertiliser control 2.5 g/L	8.71 ± 0.10

Values represent mean ± SD of analytical triplicates (*n* = 3).

### 3.3 *Effect of* var*ying application methods and varying doses of StimBlue^+^* on phytochemical composition and antioxidant capacity of *D. Suffruticosum* leaf tissue

**Figure 1:**
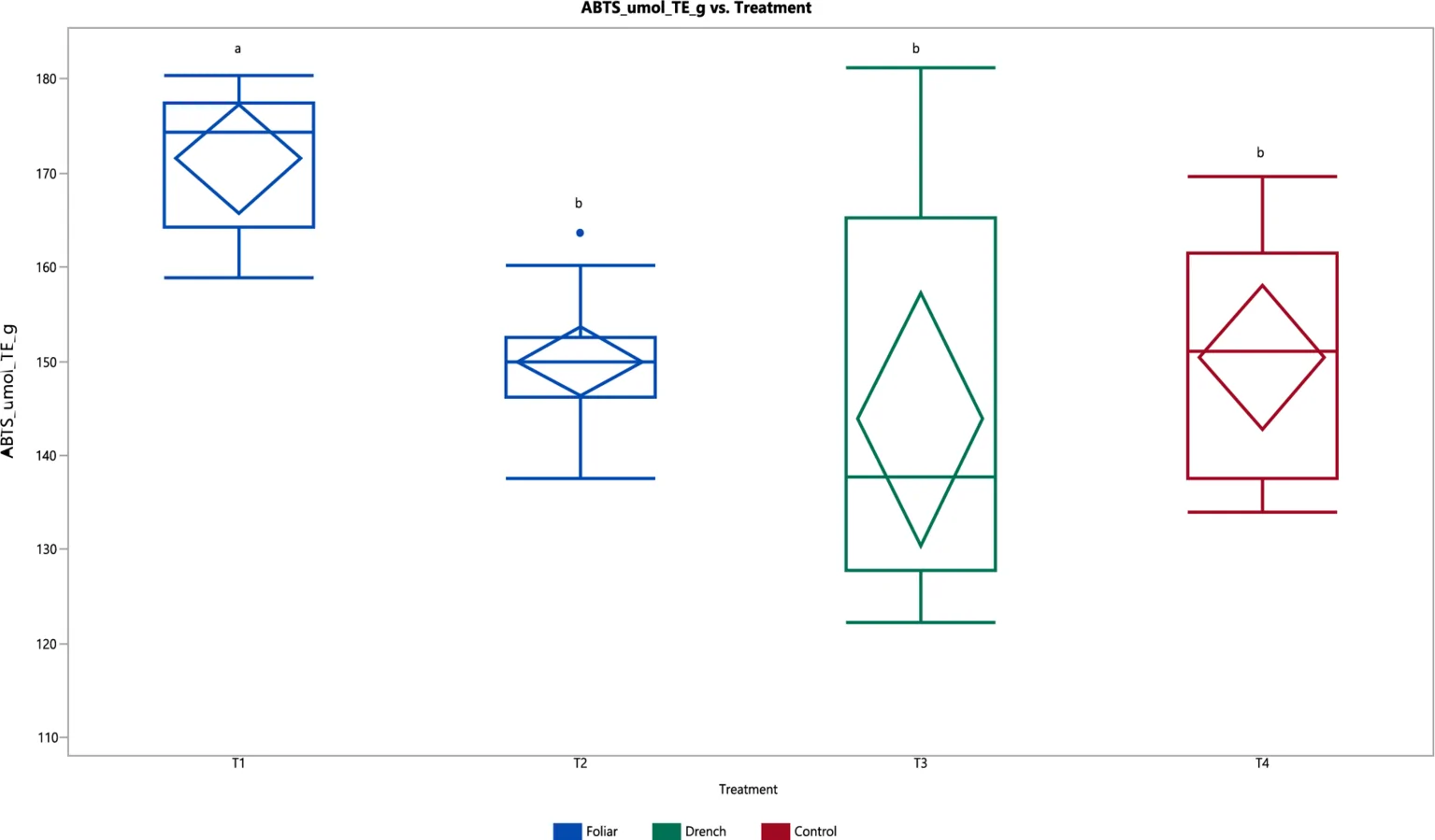
Box plots showing ABTS radical scavenging activity (μmol Trolox equivalents per gram, μmol TE/g) across four treatment groups (T1–T3) applied as foliar and drenching application methods and control (T4). T1 exhibited significantly higher antioxidant capacity compared to T2, T3, and T4 (all labelled b), indicating that foliar application at the T1 rate most effectively enhanced ABTS-based antioxidant activity. Letters above boxes denote statistically significant differences (*p* < 0.05).

**Figure 2:**
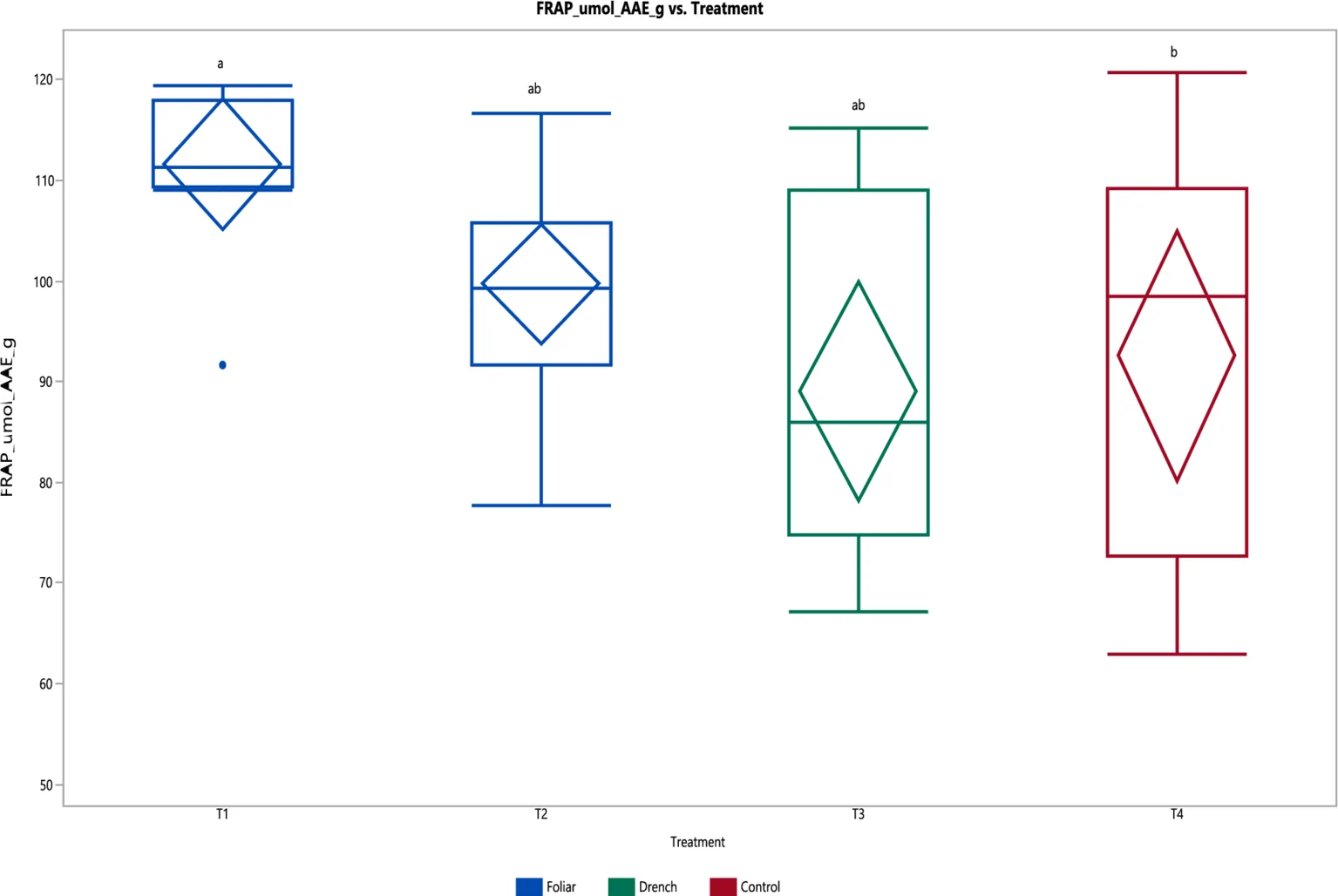
Box plots depicting ferric reducing antioxidant power (FRAP, μmol ascorbic acid equivalents per gram, μmol AAE/g) across treatment groups (T1–T3) delivered via foliar and drenching application methods and control (T4). T1 showed the highest FRAP values (labelled a), while T4 (control) recorded the lowest (labelled b); T2 and T3 were intermediate (labelled ab). Letters above boxes indicate statistically significant groupings (*p* < 0.05).

**Figure 3:**
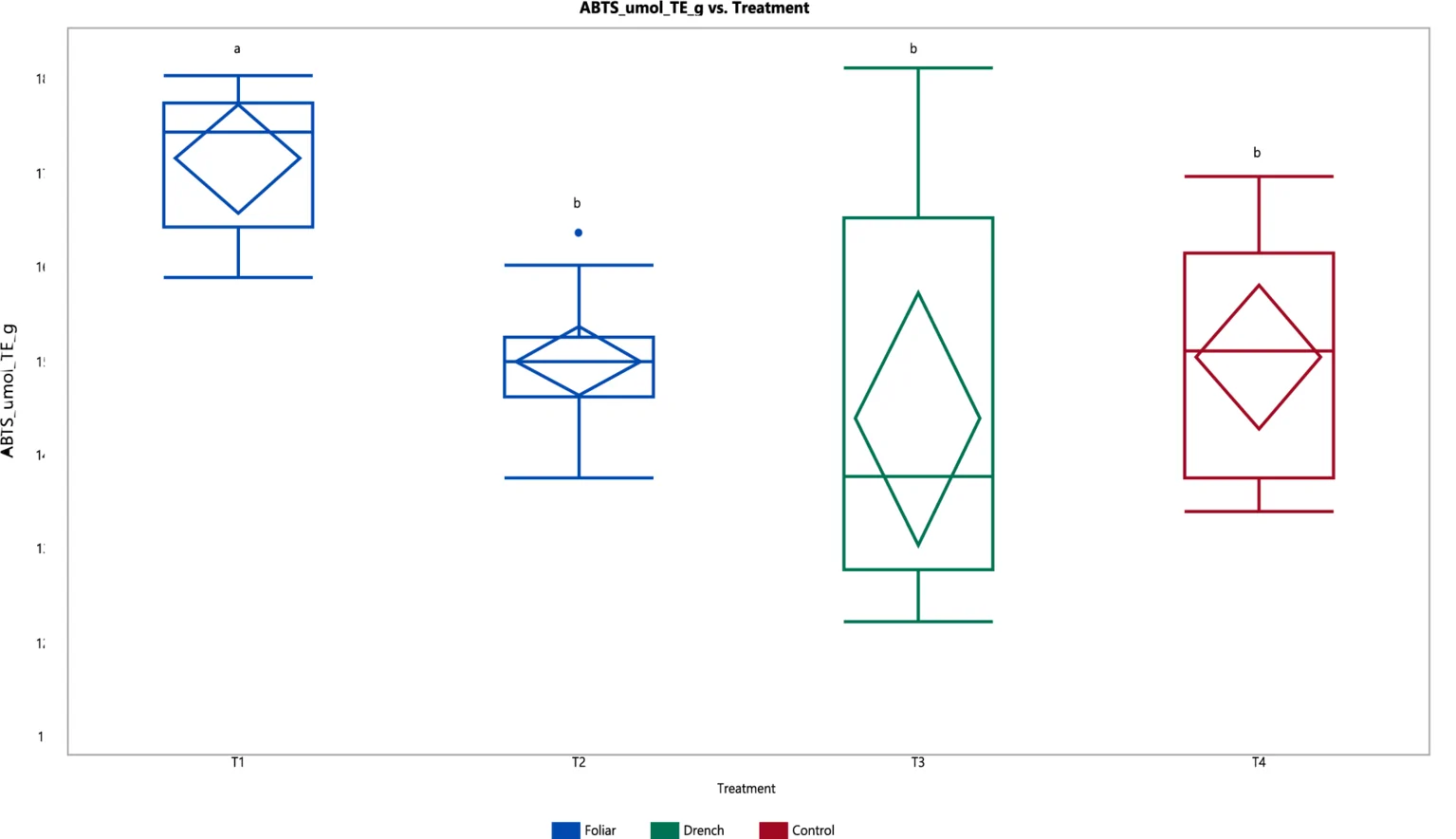
Box plots illustrating DPPH free radical scavenging activity (μmol Trolox equivalents per gram, μmol TE/g) for treatment groups T1–T3 delivered via foliar and drenching application methods and T4 as a control. T1 demonstrated significantly greater DPPH antioxidant activity than all other treatments (labelled b), suggesting that foliar delivery at this concentration most strongly inhibited DPPH radicals. Letters above boxes denote statistically significant differences (*p* < 0.05).

**Figure 4:**
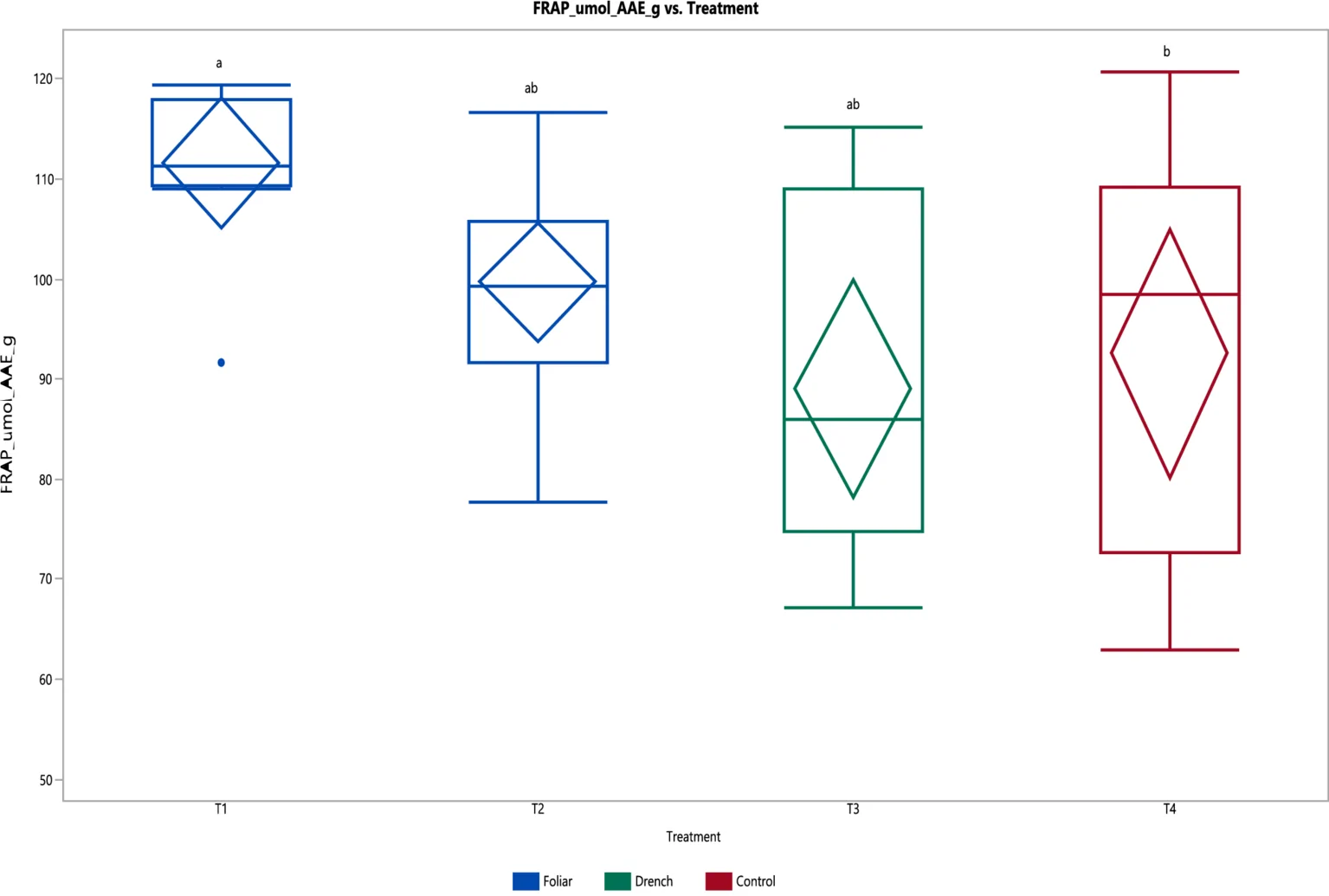
Box plots presenting total polyphenol content (μmol gallic acid equivalents per gram, μmol GAE/g) across treatment groups T1–T3 applied via foliar and drenching application methods, and T4 as a control. No statistically significant differences were detected among treatments (all groups labelled a), indicating that application method and rate did not significantly influence total polyphenol accumulation. Letters above boxes denote treatment groupings based on post-hoc analysis (*p* < 0.05).

The effects of StimBlue^+^ application on total phenolic content (TPC) and antioxidant capacity are presented in Figures 1–4. Treatment significantly affected ABTS radical scavenging capacity (*p* = 0.0002), DPPH radical scavenging activity (*p* = 0.0003), and ferric reducing antioxidant power (FRAP) (*p* = 0.0135), whereas TPC was not significantly influenced (*p* = 0.0593). Overall, the lowest foliar StimBlue^+^ application rate (T1; 2.5 mL/L consistently produced the highest antioxidant activity across all assays and was significantly greater than the fertiliser control for ABTS, DPPH and FRAP. Although treatment effects on TPC were not statistically significant, T1 exhibited the highest phenolic content (20.41 ± 1.2 mg GAE /g representing a 28.2% increase over the fertiliser control (16.0 ± 1.0 mg GAE/g) (Figures 1–4).

### 3.4 *Effect of* var*ying application methods and varying doses of StimBlue^+^* on phytochemical composition and antioxidant capacity of *D. Suffruticosum* root tissue

The phytochemical composition and antioxidant capacity of *D. suffruticosum* root tissue are presented in Table 6 as means ± SD of analytical triplicates derived from four composite biological samples. Overall, T1 (2.5 mL L^−1^ foliar application) consistently recorded the highest values across all phytochemical and antioxidant assays, whereas the fertiliser control (T4) produced the lowest values. T3 (7.0 mL L^−1^ soil drench) generally produced intermediate responses, while T2 (7.5 mL L^−1^ foliar application) showed values comparable to the control for DPPH and total phenolic content. Similar response patterns were observed in both leaf and root tissues (Table 6).

## 4 Discussion

Given that biostimulants are recognised for their stimulatory effects on many agricultural and economically important plants (11, 12). The present study demonstrated that StimBlue^+^ positively influenced *Dasispermum suffruticosum*, although the magnitude of the response depended on the application method and dosage. Compared with the fertiliser control, plants treated with the 7.0 mL L^−1^ growing-medium drench exhibited greater plant height, leaf number and chlorophyll content (Tables 4, 5). These findings are consistent with the recognised role of seaweed extract (SWE) biostimulants in stimulating plant growth and improving crop performance (11, 12).

Similar responses have been reported in *Solanum lycopersicum* (13), *Vigna unguiculata* (14) and *Abelmoschus esculentus* treated with Kelpak® SWE (15). This islikely ascribed to the diverse bioactive compounds naturally present in brown SWE, including cytokinins, auxins, gibberellins, betaines and polysaccharides. These compounds regulate cell division, cell expansion and nutrient mobilisation while stimulating root activity and photosynthetic performance, ultimately promoting plant growth and biomass accumulation (16). The superior performance of the growing-drench treatment suggests that root-zone application may have improved the availability and utilisation of these bioactive compounds, thereby enhancing plant physiological processes throughout the growth period. Similarly, Gowtham et al. (17) reported a significant increase in stem diameter following drench application of strain M9 biostimulant relative to the untreated control, consistent with the stem diameter enhancement observed under drench treatments in the present study (Table 4) This indicates that root-applied biostimulants may improve structural development through enhanced nutrient acquisition and carbon allocation Table 7.

**Table 7:** Antioxidant capacity of *D. suffruticosum* root tissue in response to StimBlue⁺ application methods and doses.

Treamentt	Treatment description	ABTS (μmol TE/g DW)	FRAP (μmol AAE/g DW)	DPPH (μmol TE/g DW)
T1	Foliar spray 2.5 mL/L	122.90 ± 1.50	63.62 ± 7.61	43.77 ± 2.29
T2	Foliar spray 7.5 mL/L	107.12 ± 1.17	53.84 ± 8.33	41.68 ± 2.67
T3	Soil drench 7.0 mL/L	111.68 ± 2.83	50.93 ± 3.38	36.09 ± 3.23
T4	Fertiliser control 2.5 g/L	92.42 ± 0.99	43.20 ± 8.99	30.23 ± 1.82

Values represent mean ± SD of analytical triplicates (*n* = 3).

Chlorophyll content was also significantly influenced by StimBlue^+^ application, with plants treated with the 7.0 mL/L growing medium drench recording the highest chlorophyll concentration of 1.30 mg/m^2^ (Table 5). Chlorophyll is fundamental for light interception and photosynthetic carbon assimilation; therefore, increased chlorophyll content reflects an enhanced photosynthetic capacity that can support greater vegetative growth (18, 44). SWE biostimulants are known to stimulate chlorophyll biosynthesis while reducing chlorophyll degradation, particularly through the activity of cytokinins and betaines that delay leaf senescence and maintain pigment stability under both favourable and stressful conditions (45–48). Similar increases in chlorophyll content following SWE application have been reported by Aremu et al. (15), Ali et al. (7), Hamouda et al. (19), Stasio et al. (49), Raja and Vidya (20), and Mughunth et al. (21). Root number was not significantly influenced by treatment, suggesting that StimBlue^+^ primarily enhanced shoot growth rather than stimulating the initiation of additional roots during the experimental period. Nevertheless, improvements in above-ground growth do not necessarily require increases in root number. SWE biostimulants have frequently been shown to improve root function, nutrient uptake efficiency and rhizosphere activity without substantially increasing root biomass (15). Consequently, the improved vegetative performance observed in this study may reflect enhanced root physiological activity rather than changes in root quantity alone.

The mineral composition of *D. suffruticosum* further supports the physiological effects of StimBlue^+^. Although the fertiliser control produced the highest N, P and S concentrations of in both leaves and roots, this response was expected because synthetic fertilisers directly supply these macronutrients in readily available forms. Rather than acting as nutrient sources, SWE biostimulants primarily improve nutrient acquisition, assimilation and utilisation efficiency within the plant (32, 15). These findings are consistent with those of Layek et al. (50) and Ertani et al. (51), who reported enhanced uptake of N, P, and in *Zea mays* L. and *Oryza sativa, respectively* following the application of SWE biostimulants combined with nutrient solutions or NPK fertiliser.

A notable increase in leaf K, Ca, and Mg compared with the control in both foliar and drenched plants from T3-T5 was observed in the present study. These minerals play essential physiological roles that likely contributed to the improved plant performance observed. K regulates osmotic adjustment, enzyme activation and stomatal function, thereby supporting photosynthesis and water-use efficiency. Calcium contributes to membrane stability, cell wall integrity and intracellular signalling during stress responses, whereas magnesium forms the central atom of the chlorophyll molecule and is therefore indispensable for photosynthetic metabolism (22). The greater accumulation of these minerals under StimBlue^+^ application may therefore explain the enhanced chlorophyll content and vegetative growth observed in the treated plants. Similar improvements in K accumulation in turnip grown under nutrient-stress conditions following SWE application have been reported by Stasio et al. (49).

The accumulation of several metallic micronutrients, including leaf and root Fe (244 and 1800 mg/kg), and Mn (266 and 400 mg/k), increased following StimBlue^+^ application (Table 6). These elements serve as essential cofactors for numerous enzymes involved in photosynthesis, respiration, and antioxidant metabolism. Improved micronutrient status may therefore have supported the biosynthesis of secondary metabolites and strengthened the plant antioxidant defence system. Similar increases in micronutrient accumulation following seaweed extract application have been reported by Mannino et al. (10). Notably, Na and Fe concentrations were markedly elevated in both leaf and root tissues across all treatments (Table 6). This finding is consistent with that reported by Sogoni et al. (52) and is attributed to the halophytic nature of *D. suffruticosum* rather than a biostimulant-driven response.

The concurrent increases in total phenolic content and antioxidant activity observed in the present study indicate that StimBlue^+^ enhanced the antioxidant defence system of *D. suffruticosum*. The positive association between phenolic concentration and the ABTS, FRAP, and DPPH assays is consistent with previous reports demonstrating that phenolic compounds substantially contribute to plant antioxidant capacity (23, 24). These responses may result from bioactive polysaccharides within SWE also present in StimBlue^+^, acting as elicitors that stimulate phenylalanine ammonia-lyase (PAL), a key regulatory enzyme in the phenylpropanoid pathway responsible for phenolic biosynthesis (25, 26). Similar improvements in antioxidant capacity following SWE application have also been reported by Mannino et al. (27).); however, antioxidant activity (measured by ABTS and DPPH) was not significantly correlated with total phenolic content (TPC), unlike the positive association observed in the present study and by Arikan-Algul et al. (23). Collectively, these findings indicate that seaweed extract biostimulants consistently enhance plant antioxidant capacity, although the contribution of phenolic compounds to this response may vary depending on species, environmental conditions and biostimulant composition.

In contrast, StimBlue^+^ treatments promoted greater accumulation of calcium, potassium, magnesium and several micronutrients than the fertiliser control. These minerals play essential physiological roles that likely contributed to the improved plant performance observed. Potassium regulates osmotic adjustment, enzyme activation and stomatal function, thereby supporting photosynthesis and water-use efficiency. Calcium contributes to membrane stability, cell wall integrity and intracellular signalling during stress responses, whereas magnesium forms the central atom of the chlorophyll molecule and is therefore indispensable for photosynthetic metabolism (22). The greater accumulation of these minerals under StimBlue^+^ application may therefore explain the enhanced chlorophyll content and vegetative growth observed in the treated plants. Similar improvements in potassium accumulation following SWE application have been reported by Stasio et al. (49).

The elevated sodium concentrations observed across all treatments are characteristic of the halophytic nature of *D. suffruticosum* and are therefore unlikely to represent a direct response to StimBlue^+^ application. Sogoni et al. (52) similarly reported naturally high sodium accumulation in this species as an adaptive mechanism that enables growth in saline coastal environments.

The concurrent increases in total phenolic content and antioxidant activity observed in the present study indicate that StimBlue^+^ enhanced the antioxidant defence system of *D. suffruticosum*.

Overall, the findings indicate that StimBlue^+^ functions primarily as a physiological biostimulant rather than as a direct nutrient source. By improving nutrient acquisition, chlorophyll accumulation and antioxidant metabolism, StimBlue^+^ enhanced the growth and physiological performance of *D. suffruticosum*. The superior responses observed under low-dose foliar application for phytochemical traits and intermediate soil drench application for vegetative growth highlight the importance of optimising both application method and dosage when using seaweed-derived biostimulants for the cultivation of underutilised indigenous crops.

## 5 Conclusion

In conclusion, growing medium-drench at 7.0 mL/L optimised vegetative growth, whilst foliar spray at 2.5 mL/L demonstrated superior efficacy for root development and phytochemical quality. All the treatments selectively elevated K, Ca, Mg, Fe, Mo, and Zn above the fertiliser control. Overall, the findings indicate that StimBlue^+^ acts primarily as a physiological biostimulant rather than a nutrient source. By improving nutrient acquisition, chlorophyll accumulation and antioxidant metabolism, particularly under low-dose foliar application (2.5 mL/L) and intermediate soil drench application, StimBlue^+^ enhanced the growth and physiological performance of *D. suffruticosum*. These responses demonstrate the potential of StimBlue^+^ and general SWE biostimulants to improve the cultivation of underutilised indigenous species while supporting sustainable production systems.

## Data Availability

The original contributions presented in the study are included in the article/supplementary material, further inquiries can be directed to the corresponding author.
